# Scientific Reasoning in Plastic Surgery

**DOI:** 10.1055/a-2952-9547

**Published:** 2026-10-05

**Authors:** Hyunsuk Peter Suh

**Affiliations:** 1 Department of Plastic and Reconstructive SurgeryAsan Medical Center, University of Ulsan College of MedicineSeoulKorea (the Republic of)

**Figure:**
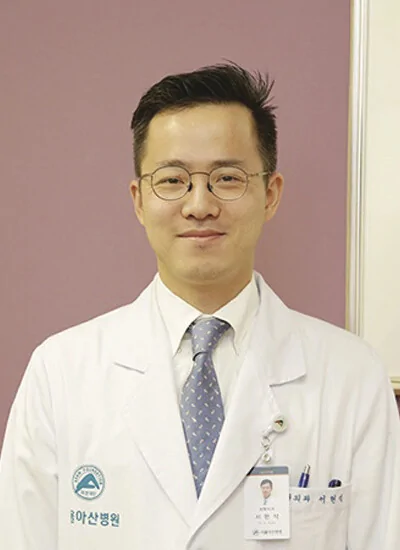
Editor-in-Chief Archives of Plastic Surgery

Plastic surgery has not always depended on rigorous scientific reasoning in the same way as other fields of medicine. Much of the early literature in the specialty focused on anatomical studies, case reports, introductions of new techniques, or simple before-and-after photographs in aesthetic surgery. These contributions were valuable, but they relied mostly on description and demonstration, rather than on hypothesis-driven research or structured analysis. By contrast, most areas of clinical medicine demand systematic reasoning—identifying important variables, recognizing bias, and carefully interpreting data. In fact, in surgery and medicine, we often need to think *more scientifically than science itself.*

In the natural sciences, discoveries often come from theory or mathematics, where equations can predict how things behave. Medicine is different. Even when a hypothesis makes sense, the results in real patients can be very different. Human biology is complex, and social or institutional factors further complicate outcomes.

History shows that even the claims of very intelligent or famous people can turn out to be wrong or based on weak evidence. Medicine is no exception. A single observation or one case report cannot prove anything. In plastic surgery, where most studies are retrospective, the risks of bias and error are especially high. For example, do we truly have enough evidence about the effect of lymphovenous anastomosis for lymphedema? Is the evidence strong enough for the surgical management of migraine? These questions remain open.

One of the greatest problems in clinical research is leaving out key variables. If important factors are missing, conclusions can be misleading. At times, small and less meaningful variables may appear statistically significant. For example, consider human height. We know that genetics, diet, and sleep are all factors, but twin studies have shown a genetic correlation of 0.97—almost proving that heredity is the dominant factor. 1 Yet some studies have compared average national height with gross domestic product (GDP) and found a correlation. 2 Does that mean GDP truly determines height? Of course not. The mistake was not including genetics as a variable. In the same way, in medical research, statistical significance without complete variable analysis can lead to the wrong conclusion, no matter how convincing it looks.

Bias is also present outside of study design. Surgeons themselves face what might be called “surgeon's bias.” The choice of surgical method may be influenced not only by training but also by the type of hospital, the payment system, or financial incentives. A surgeon may even choose technically impressive but high-risk procedures to gain recognition, to publish striking results, or to present eye-catching photos at a meeting. In such cases, the information shared may be exaggerated or unconsciously distorted.

For these reasons, as readers of the literature or audiences at conferences, we must always be critical. The weaknesses of plastic surgery research are clear. Most studies are retrospective, often lack control groups, and almost never include sham operations. Unlike pharmacology, where placebo-controlled trials are standard, plastic surgery has rarely considered the power of the placebo effect. Yet the impact of a skin incision alone, even without further action, could be far stronger than a placebo pill. Without acknowledging this, we cannot know the true benefit of our interventions.

It is also important to remember that the “level of evidence” does not depend only on whether a study is prospective or controlled. High-quality evidence requires thoughtful design *and* conscious efforts to reduce bias. Plastic surgery may never reach the same level of scientific certainty as other fields because of its variability and reliance on surgical artistry. But progress depends on raising the standard of evidence and applying scientific reasoning as much as possible.

The pursuit of rigor in plastic surgery is not about perfection. It is about consistently working to recognize bias, include meaningful variables, and interpret evidence with humility. Scientific reasoning in our specialty may never be flawless, but every step toward greater rigor makes the field stronger.
